# Supplementary material for: Prilling as an Effective Tool for Manufacturing Submicrometric and Nanometric PLGA Particles for Controlled Drug Delivery to Wounds: Stability and Curcumin Release
Source: Pharmaceutics. 2025 Jan 17;17(1):129. doi: 10.3390/pharmaceutics17010129 (PMC11768656; doi:10.3390/pharmaceutics17010129)
Supplement: Supplementary file 1 [file pharmaceutics-17-00129-s001.zip › pharmaceutics-3403243-supplementary.pdf]

Supplementary Materials

Prilling as an Effective Tool for Manufacturing Submicrometric and Nanometric PLGA Particles for Controlled Drug Delivery to Wounds: Stability and Curcumin Release

Chiara De Soricellis, Chiara Amante, Paola Russo, Rita Patrizia Aquino and Pasquale Del Gaudio

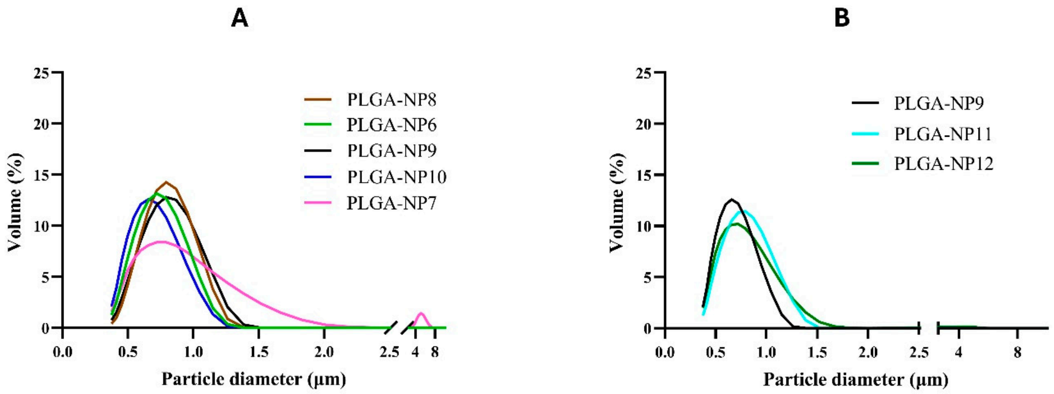

Figure S1. (**A**)Different concentrations of Tween85 (3.5; 3; 2.5;1.5; 0.5 % *v*/*v*); (**B**) PLGA dissolved in EtOAc, PVA$_{LMW}$ 0.5% (*w*/*v*) and the influence of three different nozzles diameter (80; 120; 200 μm).

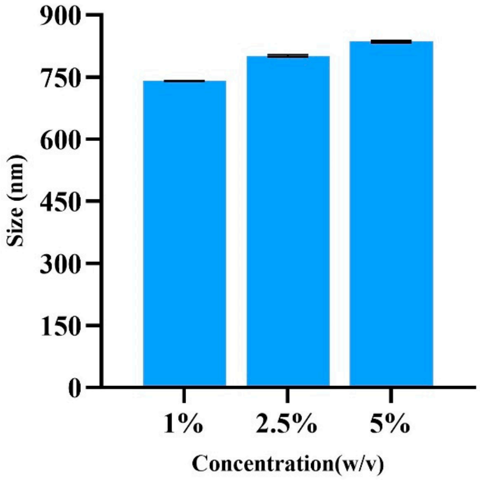

Figure S2. Effect of PLGA concentration.

Table S1. d$_{50}$ and SD (±) values of colloidal suspension obtained using different concentrations of PLGA.

PLGA Concentration (w/v)	d$_{50}$ (μm)	SD (±)
PLGA 1%	0.757	0.580
PLGA 2.5%	0.801	2.520
PLGA 5%	0.836	1.000

Table S2. d_{50} and SD (±) values of colloidal suspension obtained using different operating parameters.

**Code**	**d_{50} (μm)**	**SD (±)**
PLGA-NP1	1.190	0.023
PLGA-NP2	1.120	-
PLGA-NP3	0.870	0.003
PLGA-NP4	0.787	0.003
PLGA-NP5	0.796	0.007
PLGA-NP6	0.726	0.002
PLGA-NP7	0.839	0.012
PLGA-NP8	0.800	0.001
PLGA-NP9	0.757	0.002
PLGA-NP10	0.757	0.001
PLGA-NP11	0.774	0.001
PLGA-NP12	0.744	0.002
